# Physicochemical properties and in vitro cytotoxicity of iron oxide-based nanoparticles modified with antiangiogenic and antitumor peptide A7R

**DOI:** 10.1007/s11051-017-3859-x

**Published:** 2017-04-26

**Authors:** Anna Niescioruk, Dorota Nieciecka, Anna K. Puszko, Agata Królikowska, Piotr Kosson, Gerard Y. Perret, Pawel Krysinski, Aleksandra Misicka

**Affiliations:** 1 Faculty of Chemistry, University of Warsaw, Pasteura 1, 02-093 Warsaw, Poland; 2 Department of Neuropeptides, Mossakowski Medical Research Centre, Polish Academy of Sciences, Pawinskiego 5, 02-106 Warsaw, Poland; 3 Sorbonne Paris Cité, Université Paris 13, INSERM U1125, 74 rue Marcel Cachin, 93017 Bobigny, France

**Keywords:** Superparamagnetic nanoparticles, SPIONs, Angiogenesis, A7R peptide, Neuropilin-1, Health effects

## Abstract

Superparamagnetic iron oxide-based nanoparticles (SPIONs) are promising carriers as targeted drug delivery vehicles, because they can be guided to their target with the help of an external magnetic field. Functionalization of nanoparticles’ surface with molecules, which bind with high affinity to receptors on target tissue significantly facilitates delivery of coated nanoparticles to their targeted site. Here, we demonstrate conjugation of an antiangiogenic and antitumor peptide ATWLPPR (A7R) to SPIONs modified with sebacic acid (SPIONs-SA). Successful conjugation was confirmed by various analytical techniques (FTIR, SERS, SEM-EDS, TEM, TGA). Cell cytotoxicity studies, against two cell lines (HUVEC and MDA-MB-231) indicated that SPIONs modified with A7R reduced HUVEC cell viability at concentrations higher than 0.01 mg Fe/mL, in comparison to cells that were exposed to either the nanoparticles modified with sebacic acid or A7R peptide solely, what might be partially caused by a process of internalization.

**Electronic supplementary material:**

The online version of this article (doi:10.1007/s11051-017-3859-x) contains supplementary material, which is available to authorized users.

## Introduction

Cancer is one of the major health concerns worldwide. It is the second most common cause of death in the USA, following heart disease (Siegel et al. 2014). Cancer results from chromosomal, genetic, or epigenetic events, leading to several cellular abnormalities like uncontrolled proliferation, increased resistance to apoptosis, replicative immortality, and metabolic shift reinforced by disorders like local increased angiogenesis and immune tolerance (Hanahan and Weinberg 2011). One of the processes which play a crucial role in solid tumor growth is angiogenesis (Folkman 1971). In this process, new capillaries are generated from pre-existing blood vessels, which allow the delivery of oxygen and nutrients for tumor cells and facilitate tumor progression, invasion, and metastasis. Therefore, antiangiogenic therapy is one of the promising approaches for cancer treatment (Samant and Shevde 2011). Among various proangiogenic factors, the most essential and characterized is vascular endothelial growth factor A (VEGF A) (McMahon 2000). The VEGF A’s predominant isoform is VEGF_165_ which binds with high affinity to tyrosine kinase domain receptors (VEGFRs) on the surface of endothelial cells (Ferrara et al. 2003). VEGF_165_/VEGFR complex formation is a critical step, which induces the major proangiogenic activities of VEGF, including cell proliferation, migration, and capillary sprouting (Dvorak 2002). This ligand-receptor binding can be significantly enhanced by association of VEGFR with the co-receptor neuropilin-1 (NRP-1) (Soker et al. 1998) This transmembrane glycoprotein is also widely expressed in tumor cells, such as lung, stomach, colon, breast, pancreatic, and glioma cancers (Chaudhary et al. 2014), suggesting its role in increased vascularity, tumor progression, aggressiveness, and meaning poor prognosis (Lu et al. 2015).

Nanotechnology is a growing field that offers promising applications for cancer detection, diagnosis, and treatment (Jabir et al. 2012). In recent years, the superparamagnetic iron oxide-based nanoparticles (SPIONs) have emerged as an attractive, potential theranostic tool in biomedical applications and diagnostics, including magnetic resonance imaging (MRI), induced hyperthermia cancer treatment, and drug delivery (Sun et al. 2008; Veiseh et al. 2010). SPIONs exhibit a wide variety of properties which make them highly promising carriers for a drug delivery. The intrinsic magnetic properties of SPIONs (due to ferromagnetic iron) allow the remote control of their accumulation by means of an external magnetic field (Arruebo et al. 2007). Development of this magnetic delivery system mandates that the SPIONs behave magnetic only under the influence of an external magnetic field, but not when this field is removed (Mody et al. 2014). In addition, high surface area-to-volume ratio and the ability to chemically modify nanoparticles surface with various functional groups allows them to covalently bind different molecules to their surface. Functionalization of nanoparticles’ surface with targeting molecules such as peptides (Hansen et al. 2013), proteins (Shevtsov et al. 2015), and antibodies (Ota et al. 2014) facilitates delivery of SPIONs to tumor tissues via active targeting (Bertrand et al. 2014). Such a multi-purposed delivery system (active and magnetic targeting) might result in much more effective delivery of nanoparticles to tumors.

The use of peptides as targeting ligands has a number of advantages in terms of low toxicity, high selectivity, and potency (Fosgerau and Hoffmann 2015). They are smaller than protein ligands and can be easily synthesized and modified at large scale and conjugated to other molecules. It has been shown that some peptides can bind to integrins, overexpressed receptors on tumor cells or tumor-associated blood vessels (Boohaker et al. 2012). Therefore, conjugation of imaging agents, small-molecule drugs, or nanoparticles to tumor targeting peptides increase specificity and efficacy of their delivery to cancer tissues and reduce non-specific toxic effect (Laakkonen and Vuorinen 2010; Zhang et al. 2012). One of the promising peptides that can be used not only as a targeting ligand but also as antiangiogenic molecules is a heptapeptide ATWLPPR (A7R), which binds specifically to NRP-1 and selectively inhibits VEGF_165_ binding to this receptor (Binétruy-Tournaire et al. 2000). In vitro and in vivo studies have shown that A7R inhibits tumor growth and angiogenesis in a breast cancer cell xenograft in nude mice (Starzec et al. 2006). A few reports have shown that peptide sequence ATWLPPR may be used to functionalize surface of gadolinium (Benachour et al. 2012), gold (Bartczak et al. 2013), and silica nanoparticles (Ciccione et al. 2016).

In this work, we synthesized SPIONs of controlled surface chemistry for the purpose of the formation of antiangiogenic peptide/SPION conjugate. Due to the magnetic properties of SPIONs, such a multi-purpose conjugate can be effectively guided to and maintained within the area of tumor with the help of an external magnetic field, whereas the peptide A7R ligand inhibits angiogenesis by interaction with specific receptors (NRP-1) located in a large amount on the surface of cancer cells.

## Experimental

### Chemicals

All chemicals were of the highest quality available and were used without further purification. Fmoc-Arg(Pbf)-Wang resin (0.39 mmol/g), Fmoc-Leu-OH, Fmoc-Trp(Boc)-OH, Fmoc-Thr(*t*Bu)-OH, Fmoc-Ala-OH, O-(Benzotriazol-1-yl)-*N,N,N′,N′*-tetramethyluronium tetrafluoroborate (TBTU), and 6-chloro-1-hydroxybenzotriazole (6-Cl-HOBt) were purchased from Activotec. Fmoc-Pro-OH was purchased from Iris Biotech GmbH. Dimethylformamide (DMF), dichloromethane (DCM), trifluoroacetic acid (TFA), triisopropylsilane (TIS), piperidine, phenol (PhOH), acetonitrile, *N*,*N*-diisopropylethylamine (DIPEA), nitrate salts of iron(III), nickel, zinc, potassium bromide, sebacoyl chloride, Dulbecco’s modified Eagle medium (DMEM), and hydroxylamine hydrochloride (99.9%) were purchased from Sigma-Aldrich. 4-Methylmorpholine was purchased from Fluka. 1-Ethyl-3-(3-dimethylaminopropyl)carbodiimide hydrochloride (EDC) was purchased from Merck. Isopropanol (IPA), diethyl ether (Et_2_O), acetone, ethanol, nitric acid, hydrochloric acid, silver nitrate, and sodium hydroxide were purchased from POCH. Flat-bottom sterile polystyrene 96-well plates were purchased from TPP. Sterile phosphate buffer saline (PBS) was purchased from Lonza. Human umbilical vein endothelial cells (HUVECs) were purchased from ATCC, LGC Standards. MDA-MB-231 and endothelial cell growth medium (EGM) were purchased from PromoCell. MTS (3-(4,5-dimethylthiazol-2-yl)-5-(3-carboxymethoxyphenyl)-2-(4-sulfophenyl)-2H-tetrazolium) was purchased from Promega. Fetal bovine serum (FBS) and l-glutamine were purchased from Gibco. Penicillin and streptomycin were purchased from Biological Industries. All aqueous solutions were prepared with Mili-Q water.

### Synthesis of SPIONs-SA

Superparamagnetic nanoparticles, with the general formula Ni_0.5_Zn_0.5_Fe_2_O_4_, were synthesized by co-precipitation procedure described previously (Majewski and Krysinski 2008; Brzozowska and Krysinski 2009). The synthesis consisted of mixing the heated solution of metal cations with sodium hydroxide. The precursor solution included the nitrate salts of 0.6667 mol/dm^3^ Fe^3+^, 0.1667 mol/dm^3^ Ni^2+^, and 0.1667 mol/dm^3^ Zn^2+^. To 40 mL of the precursor solution, 8 mL of 2 M HNO_3_ and 152 mL of water were added and the mixture was heated to 95 °C under reflux and with continuous stirring. In a separate container, an aqueous solution of 100 mL of 3 M NaOH and 300 mL of water was heated to 95 °C. The amount of NaOH was adjusted to obtain a final concentration of 0.3 M after precipitation of the mixed ferrites. Next, the NaOH solution was poured rapidly into the reaction vessel under vigorous stirring. The resulting dark brown precipitate was maintained at 95 °C for 12 to 16 h under stirring. The solution was cooled to room temperature, and the nanoparticles were separated with a magnet and the supernatant decanted. Then, the precipitate was washed three times with deionized water with magnet-assisted sedimentation between each wash. After synthesis, the nanoparticle surface was modified using sebacoyl chloride. First, ferrofluid was washed four times with acetone, separated with a magnet, and dried under nitrogen. The prepared sample was suspended in a dry acetonitrile under nitrogen. Then, sebacoyl chloride and 4-methylmorpholine (as Lewis base) were added to this suspension with a final volume ratio of 50:1:1, (acetonitrile/sebacoyl chloride/4-methylmorpholine). The mixture was stirred under nitrogen for 1 h. The resulting nanoparticles were separated with a magnet and washed several times with acetonitrile, ethanol, and water. Finally, the obtained nanoparticles were modified with sebacic acid (SA) due to the hydrolysis of acid chloride moiety to carboxylic group during the washing steps with water.

### Synthesis of A7R peptide

The synthesis of A7R peptide was carried out manually on the Wang resin, by the Fmoc solid-phase method, with the use of TBTU/6-Cl-HOBt as the coupling reagents, and controlling presence of a free amino group by the Kaiser or chloranil tests. The final peptide was cleaved from the resin by TFA and purified by preparative RP-HPLC using C_12_ column. Its structure was confirmed by ESI-MS. The details of peptide synthesis, purification, and characterization are described in the Supporting Information.

### Modification of magnetic nanoparticle surface with A7R peptide

**Fig. 1:**
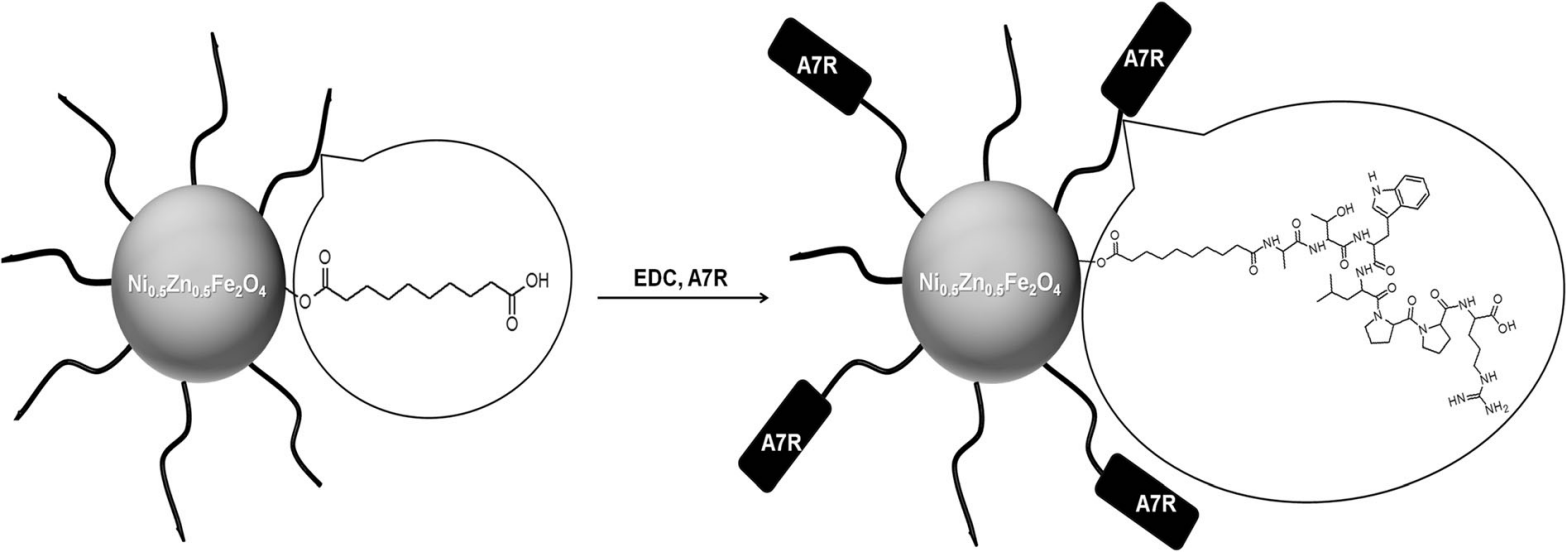
Reaction scheme of A7R peptide coupling to nanoparticles modified with sebacic acid

Conjugation of A7R peptide to magnetic nanoparticles modified with sebacic acid (Fig. 1) was performed according to the procedures given by McCarthy et al. (2012) with a slight modification. To 1 mL iron oxide-based nanoparticles (15 mg Fe/ml) suspended in a mixture of THF/H_2_O (1:1, *v*/*v*), 15.4 mg EDC in 1.54 mL THF/H_2_O (1:1, *v*/*v*) was added and the mixture was stirred for 10 min. Afterwards, 18.3 mg A7R peptide and 9 μL DIPEA in 0.65 mL THF/H_2_O (1:1, *v*/*v*) were added and the reaction mixture was stirred at room temperature for 24 h. Next, nanoparticles were collected by a magnet and supernatant over nanoparticles was discarded. Modified nanoparticles were redispersed in THF/H_2_O (1:1, *v*/*v*) and separated by a magnet for several times to remove unreacted peptide (supernatants were checked using UV-Vis spectrophotometer (SPECTROstar Nano, BMG LabTech) at 280 nm for tryptophan absorption peak. The nanoparticles were then suspended in Mili-Q H_2_O.

### Cell culture

Primary HUVECs were cultured in EGM according to manufacturer’s protocol. All used cells were from passages 2 to 4. Breast cancer MDA-MB-231 cells were maintained in DMEM supplemented with 10% FBS, 1% l-glutamine, and 1% penicillin/streptomycin.

### Cytotoxicity assay

To determine cell viability, the colorimetric MTS metabolic activity assay was used. MDA-MB-231 cells (2 × 10^4^ cells/well) and HUVEC cells (2 × 10^4^ cells/well) were seeded in a 96-well plate and grown for 48 h (37 °C, 5% CO_2_) in DMEM medium with 10% FBS and 1% l-glutamine (for MDA-MB-231) or endothelial cell growth medium (for HUVECs). After this period, HUVECs medium was exchanged for endothelial cell growth medium containing 4% FBS. Cells were incubated in the presence of nanoparticles dispersed in a sterile PBS solution, containing various final Fe concentrations (1, 10, 100, and 250 μg/ml) or peptide dissolved in PBS solution at various final concentrations (10, 100, 500, and 1000 μM). Cells treated only with medium served as a negative control group (equivalent to normal viability). The viability was determined after 1- and 4-day incubation at 37 °C in a humidified 5% CO_2_ balanced air incubator. Solution with nanoparticles was pipetted out, and wells have been washed once with medium, followed by addition of 100 μL of fresh medium before next step. Afterwards, 20 μl of MTS solution was introduced into each well. After 1 h of incubation, the absorbance intensity was measured at 490 nm (spectrophotometer SPECTROstar Nano, BMG LabTech) using a microplate reader (Cytation3, BioTek). All these experiments were performed with a single addition of nanoparticles or peptide. Each of the concentrations was tested simultaneously in quadruplicate, and each of the experiments was repeated three times. The measured absorbance was directly proportional to the number of viable cells. The relative cell viability (%) was expressed as a percentage relative to the untreated control cells.

### Characterization of conjugate

The morphology and size of the nanoparticles were analyzed by transmission electron microscope (Zeiss Libra 120EFTEM).

The elemental composition of nanoparticles with A7R peptide was examined using energy-dispersive X-ray spectroscopy analyzer (EDS) integrated with scanning electron microscope (SEM; Zeiss Merlin field emission).

The hydrodynamic size and zeta potential of nanoparticles were determined using a Malvern Instruments Zetasizer Nano ZS. Zeta potentials were measured in 10 mM NaCl.

Fourier transform infrared (FTIR) spectra in transmission mode in KBr pellets were acquired with a Nicolet 8700 spectrometer.

Normal Raman and surface-enhanced Raman scattering (SERS) spectra were collected with a Labram HR800 (Horiba JobinYvon) confocal microscope system, equipped with a Peltier-cooled CCD detector (1024 × 256 pixels). Diode-pumped, frequency-doubled Nd:YAG laser provided 532 nm excitation radiation, with a total power of less than 1 mW at the sample. Raman signals were measured in a backscattering geometry, using the holographic grating with 600 grooves per mm. For normal Raman measurements, the solids (peptide or dried unmodified and modified SPIONs) were placed on a glass microscopic slide and laser beam was focused on the sample through a ×50 Olympus objective. Typical SERS samples were prepared by mixing A7R peptide solution or A7R peptide conjugated to SPIONs (separated by magnet) with Ag NPs sol solution (respectively in 1:33 and 1:50 volumetric ratio) and measured in glass cuvettes, using a cuvette-holder. The acquisition time for a single accumulation was 60 s, but the number of the accumulations was dependent on the signal-to-noise ratio for a particular spectrum. The method of silver nanoparticle synthesis was described in the Supporting Information.

Thermogravimetric analysis was performed with a TGA Q50 (TA Instruments). The measurements were conducted under an oxygen/nitrogen atmosphere.

### Statistical analysis

Statistical analysis was performed using Prism (Version-5.01, GraphPad Software).

## Results and discussion

### Synthesis and characterization of conjugate

SPIONs were synthesized by a co-precipitation method and modified with sebacic acid as a linker. Carboxylic groups of the linker allowed conjugation of A7R peptide via the amide bond using EDC as a coupling reagent. Due to the fact that COOH group of the C-terminal arginine and four C-terminal residues (LPPR) play a crucial role in the inhibitory effect of A7R (Starzec et al. 2007), we conjugated peptide to magnetic nanoparticles by N-terminal amine group of alanine. The remaining N-terminal amino acid residues (ATW) of the A7R sequence ensure additional distance between the nanoparticle and receptor binding region (LPPR).

The magnetic properties of obtained superparamagnetic nanoparticles, with the general formula Ni_0.5_Zn_0.5_Fe_2_O_4_, were carefully examined in our previous works (Kijewska et al. 2013; Nawara et al. 2012), where we addressed in details the effect of surface modifications on magnetic properties of mixed SPIONs. In these papers, we have measured magnetization vs magnetic field for bare SPIONs, surface-modified SPIONs, and even the nanoparticles enclosed in the polymer microvessels. Only the latter system has shown a substantial decrease in the nanoparticle magnetization, but this was due to the fact that SPIONs were enclosed in a polymer shell and within the polymeric microvessel, drastically decreasing both the Brownian and Néel effect of nanoferrites. For the case of surface modifications of SPIONs (such as SPION/A7R conjugate), the overall magnetic properties of these conjugates are lower than that of the core itself and related to the amount of organic adlayer on the surface (Nawara et al. 2012). Our synthesis yielded nanoparticles of typical saturation magnetization value of magnetic core in the order of 56 emu/g (at room temperature), comparing well with the characteristics found in the literature (Millan et al. 2007; Prasad et al. 2011). Taking into account the presence of organic layer constituting ca. 35% of the total mass of the conjugate (vide infra), we expected the magnetization of SPION/A7R conjugates to be in the order of 36 emu/g (not shown), a value sufficient for utilization of their magnetic properties for targeted drug delivery. This was proved for the case of SPIONs encapsulated in polymeric microvessels, where for saturation magnetization as low as ca.18 emu/g, the vesicles migrate along the direction of the magnetic field (Kijewska et al. 2013, supporting video).

**Fig. 2:**
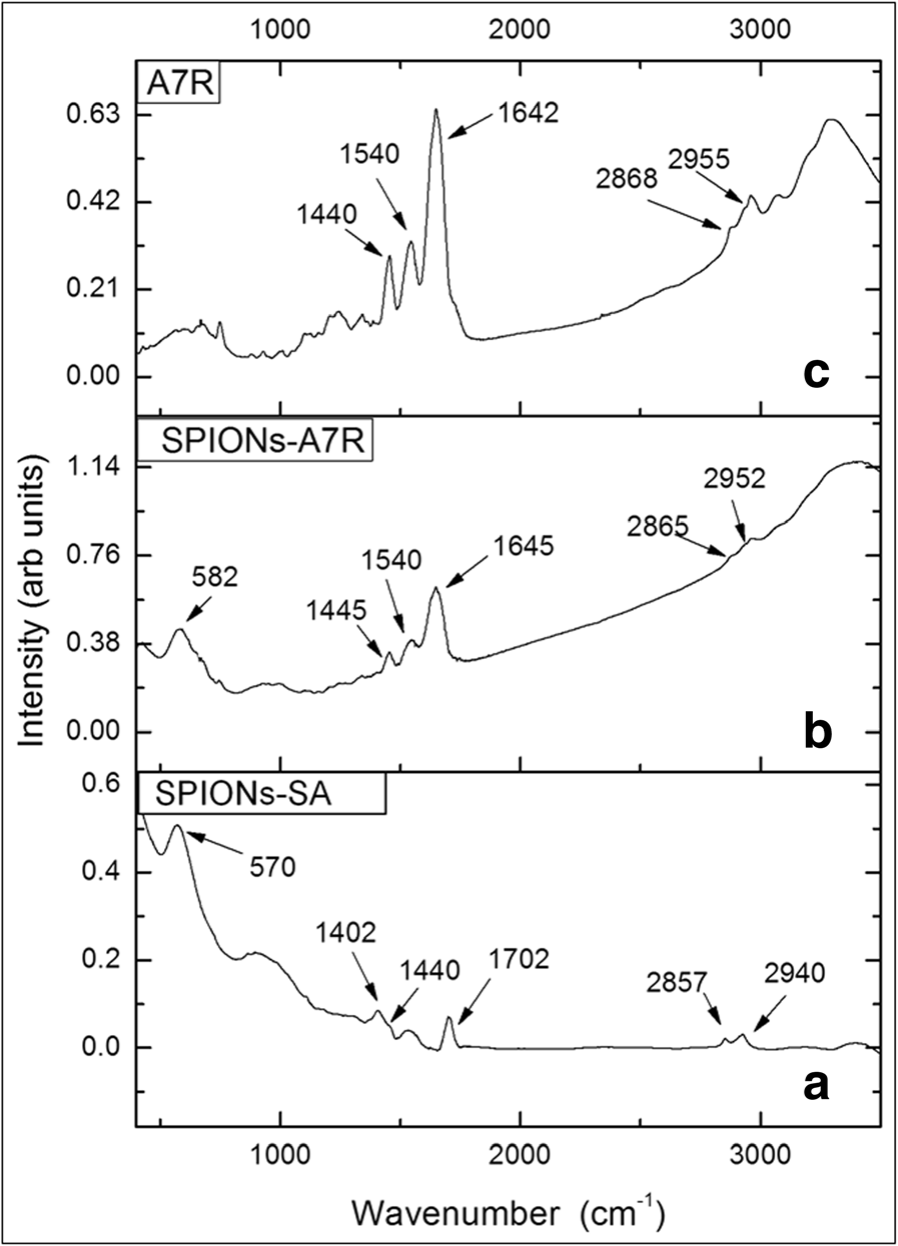
FTIR spectra of **a** SPIONs modified with sebacic acid and **b** SPIONs modified with A7R peptide (conjugate) and **c** A7R peptide

Successful modification of magnetic ferrite nanoparticles with sebacic acid (Fig. 2a) and their further conjugation with A7R peptide (Fig. 2b) was confirmed by Fourier transform infrared spectroscopy. The FTIR spectrum for SPIONs modified only by sebacic acid (Fig. 2a) exhibits band at 1702 cm^−1^, which can be ascribed to C=O stretching mode of COOH groups (Sciacca et al. 2010). Bands due to C–H stretching vibrations are observed around 2940 and 2860 cm^−1^ (Xu et al. 2016). The bands around 1440 cm^−1^ are related to C–H bending, while this around 1400 cm^−1^ can be associated with C–O–H bending or CH_2_ deformation.

All these vibrational features support an effective modification of SPIONs with sebacic acid.

The broad band around 570 cm^−1^ (Fig. 2a) can be associated with the Fe–O stretching mode (Jafari et al. 2015). This latter band is shifted to 581 cm^−1^ for SPIONs coated with peptide (Fig. 2b). IR spectrum of a solid A7R peptide (Fig. 2c) reveals intense bands at 1642 and 1540 cm^−1^, which are associated with C=O stretching (amide I) and N–H bending (amide II), respectively (Olejnik et al. 2013). The broad peak at 3330 cm^−1^ (spectra b and c) is attributed to the N–H and/or O–H stretching band of the peptide (De Palma et al. 2007). The IR spectrum of SPIONs-A7R (Fig. 2b) is similar to the peptide spectrum and shows characteristic bands at 1645 and 1540 cm^−1^, confirming the presence of the peptide onto the nanoparticle surface.

**Fig. 3:**
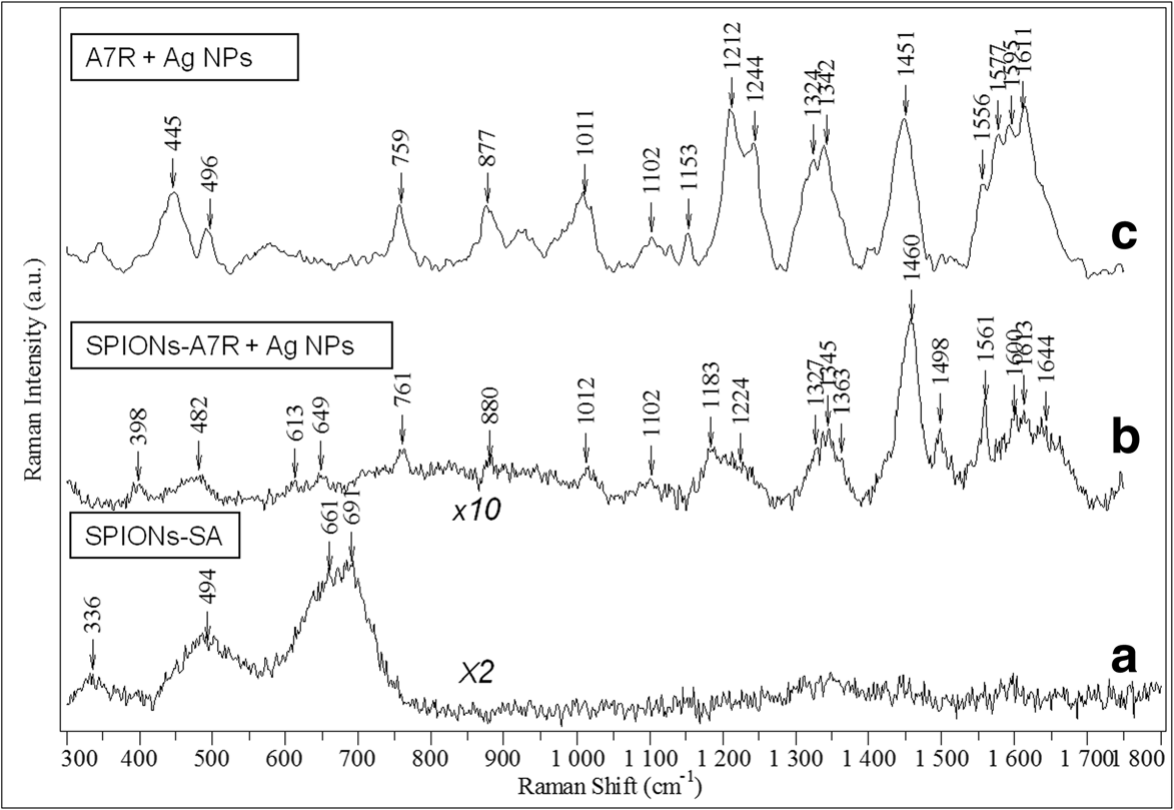
**a** Normal Raman spectrum of SPIONs modified with sebacic acid. **b** SERS spectrum of SPIONs-A7R conjugates mixed with colloidal silver nanoparticles (1:50 *v*/*v*). **c** SERS spectrum of 4 × 10^−4^ M A7R peptide aqueous solution (initial concentration) mixed with colloidal silver nanoparticles (1:33 *v*/*v*). All spectra were excited with a 532-nm laser; scaled, baselined, and shifted for the clarity of presentation. The intensity scaling factors are given as labels in the figure

Raman scattering spectroscopy was also employed to examine the efficiency of modification of magnetic nanoparticles with sebacic acid and further conjugation of A7R peptide. Unfortunately, Raman spectrum of NPs after treatment with sebacoyl chloride does not show any vibrational features of the finally formed sebacic acid. As can be seen in Fig. 3a, only the Raman active modes characteristic of a cubic spinel structure of ferrite Ni_0.5_Zn_0.5_Fe_2_O_4_ are observed (Varshney et al. 2011, Da Silva et al. 2010). It resembles strongly the vibrational pattern observed for the previously studied, identical in terms of chemical composition, but citrate stabilized superparamagnetic iron oxide-based magnetic nanoparticles (Nieciecka et al. 2016). In other words, Raman spectra of the bare SPIONs (here not shown) and after modification with sebacic acid (Fig. 3a) are nearly identical. Lack of vibrational signature of sebacic acid is related to its very low concentration in the studied sample, probably below the detection limit of normal Raman spectroscopy. Visibly broad Raman bands of the ferrite in Fig. 3a might be a signature that nanoparticles are prone to laser-induced heating upon prolonged light exposure.

Normal Raman signal of the NPs after complete attachment procedure of A7R peptide did not show any spectral signature of peptide presence (data not shown). The reason again is a low value of the Raman scattering cross-section, combined with a small amount of the examined material (A7R peptide). Ferrite NPs undergone peptide conjugation were mixed with the surface plasmon active silver nanoparticles (Ag NPs), in order to enhance the Raman scattering signal of A7R peptide and verify conjugation. This phenomenon is known as surface-enhanced Raman scattering (SERS), and the origin of the enhancement is primarily related to the local amplification of electromagnetic field, experienced by the molecules in a close proximity to plasmonic nanoparticles. The second, less significant contribution comes from the short-range chemical interactions between the molecule and nanoparticles (including light induced charge-transfer), which may modify molecular polarizability.

SERS spectrum of the A7R conjugated to magnetic nanoparticles mixed with the Ag NPs suspension, presented in Fig. 3b, shows clearly the Raman peaks, mostly due to vibrations of tryptophan (Trp) residue. The same spectral response, namely, a manifestation of the vibrations almost exclusively due to aromatic side-chain was observed in the normal Raman spectrum of solid A7R peptide (see Fig. S3 in Supporting Information) and is a quite common spectral behavior for the small peptides, containing also non-aromatic amino acids (Hernández et al. 2009). Aromatic species exhibit very high Raman cross-sections, owing to high polarizability of the delocalized electrons under laser excitation, resulting in Raman signal dominated by the vibrational bands specific for aromatic moieties. Raman spectrum of tryptophan is rather complex, and in spite of the fact it is quite well known, there is still some discrepancy in the vibrational assignment. For example, intense normal Raman mode around 880 cm^−1^ is ascribed either to H-scissoring on indole ring (Zhu et al. 2011) or vibrations of pyrrole ring (Hernández et al. 2010), while the bands at 758 and 1012 cm^−1^ are assigned to the ring breathing vibrations of indole ring by the former authors and to benzene in-plane vibrations by the latter. This proves that correct band assignment of Trp is not trivial and/or vibrational motions of both five- and six-membered rings are strongly coupled.

We conducted also a reference SERS experiment, in which an aqueous solution of non-conjugated A7R peptide was exposed to Ag NPs (see Fig. 3c). The SERS signal of the peptide interacting directly with the silver nanoparticles is quite similar to this when the peptide is believed to be conjugated to sebacic acid-modified iron oxide-based nanoparticles (compare spectra in Fig. 3c, b). Positions of the SERS bands are very similar in these two cases, also closely resembling major SERS features of the tryptophan containing dipeptide (Trp-Cys) and small-cell penetrating peptide (penetratine), adsorbed on gold nanoshells (under 785 nm excitation) (Wei et al. 2008). Most of the observed SERS bands for the sample with A7R peptide potentially conjugated to iron oxide-based nanoparticles arise from the vibrations of the tryptophan aromatic rings. SERS bands around 760 and 1012 cm^−1^ are respectively due to symmetric benzene/pyrrole in-phase and out-of-phase breathing modes (Wei et al. 2008). The features around 880 and 1560 cm^−1^ are related to indole ring vibration coupled to NH bending and C–C stretching vibration of pyrrole ring, respectively (Wei et al. 2008). There is no agreement in the literature on the origin of tryptophan SERS band around 1450 and 1460 cm^−1^ (which we observed for A7R peptide directly on Ag NPs and magnetic nanoparticles after peptide modification exposed to Ag NPs). It has been assigned to either indole ring bending and CH bend (Maiti et al. 2012), or stretching of pyrrole and benzene ring (Chuang and Chen 2009), or CH_2_ scissoring (Hussain and Pang 2015) or even stretching of a carboxylate group (Aliaga et al. 2009). Amide I band around 1660 cm^−1^, originating from polypeptide backbone, is very weak in the normal Raman spectrum of the solid A7R peptide (see Fig. S3 in Supporting Information), while it is barely visible in the SERS spectra of the free and conjugated peptide interacting with AgNPs (see the broad features around 1640 cm^−1^ in spectra b and c, Fig. 3). The suppression of amide I vibrational mode in SERS spectrum in some peptides has been already a recognized phenomenon, ascribed to distancing of the peptide bond by the presence of amino acids with bulky side chains (Kurouski et al. 2013). All SERS bands are less intense in the case of peptide covalent attachment to sebacic acid functionalized magnetic nanoparticles, which can be both due to limited interactions between the A7R peptide and Ag NPs in this form and a trace amount of the peptide itself (see the thermogravimetry results for the details, Fig. 5), comparing to SERS spectrum of A7R in aqueous solution. Summarizing, observed SERS signal confirms a successful conjugation of A7R peptide to magnetic nanoparticles. We performed a similar SERS experiment for the SPIONs conjugated only with sebacic acid, but we did not succeed to obtain any surface enhancement of the linker vibrations, probably due to its much lower Raman scattering cross-section comparing to aromatic amino acid of the peptide and/or weaker interactions with Ag NPs. The first step of the ferrite NP modification, involving sebacic acid linker attachment, is hence less clear from the SERS experiment, but it was confirmed with IR spectroscopy (see Fig. 2a).

To confirm successful conjugation of A7R peptide to SPIONs, we also used SEM-EDS analysis. The EDS spectrum of magnetic nanoparticles with A7R (Fig. S4, Supporting Information) confirms the presence of nitrogen, which is not present in magnetic nanoparticles with sebacic acid. This result indicates successful immobilization of A7R peptide onto the nanoparticle surface.

**Fig. 4:**
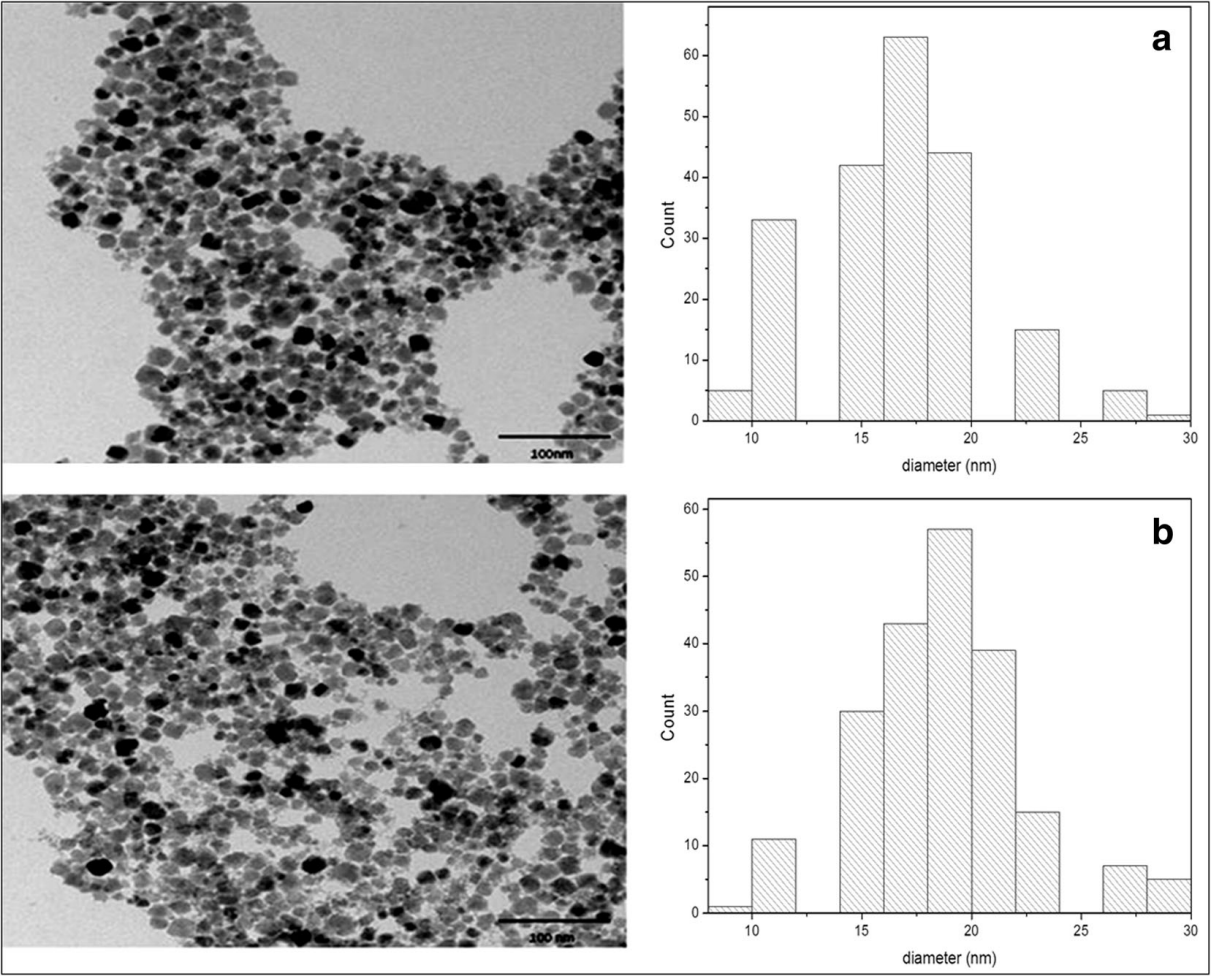
TEM images with size distribution histograms of **a** SPIONs modified with sebacic acid and **b** SPIONs with A7R peptide

The size and morphology of nanoparticles were characterized by transmission electron microscopy (TEM) and dynamic light scattering (DLS) measurements. Figure 4 shows the TEM images of magnetic nanoparticles with sebacic acid (Fig. 4a) and nanoparticles modified with peptide (Fig. 4b). Based on the analysis of TEM images, an average size of nanostructures was estimated. The average diameter of the unmodified nanoparticles is about 18 ± 0.4 nm as shown in the presented histogram (Fig. 4a). After modification with a peptide, the microscopic image of conjugates indicates that the structures are relatively uniform, with a mean size of ca. 21 ± 0.3 nm. The size distribution histograms were obtained by analyzing about 200 nanoparticles in the TEM images.

The mean size of particles was also determined by DLS technique. Since the DLS measures the so-called hydrodynamic diameter of solvated nanoparticles, the obtained value is usually higher as compared with the value obtained by TEM measurements of dry sample under vacuum conditions. An average diameter of peptide unmodified nanostructures is about 34 nm, while for conjugates, this value is three times higher and equal 115 nm. Additionally, the DLS measurements were also carried out 3 weeks after the synthesis of the conjugate. After this time, the resulting value of the diameter was still around 115 nm, which indicates the stability of nanoparticles and the lack of their aggregation—an important behavior from the point of view of potential usage of such conjugate in drug delivery platform.

In order to get deeper insight into the reason of such stability, we measured the electrokinetic potential (zeta potential) of our systems. The measured values of zeta potential for SPIONs with sebacic acid and SPIONs with A7R at pH 7 were −45.6 and −17.8 mV, respectively. The negative charge of nanoparticles originates from the presence of carboxylic groups on the surface of SPIONs covered with sebacic acid. The less negative value of zeta potential for SPIONs modified with A7R indicates an attachment of A7R to SPIONs. Such conjugates, besides carboxylic groups on the C-termini (as SPIONs modified with sebacic acid), have also protonated guanidine group of Arg at pH 7, which causes the change of zeta potential. Nevertheless, this negative value of zeta potential appears to be sufficient for stabilization of the suspension of conjugate against aggregation.

**Fig. 5:**
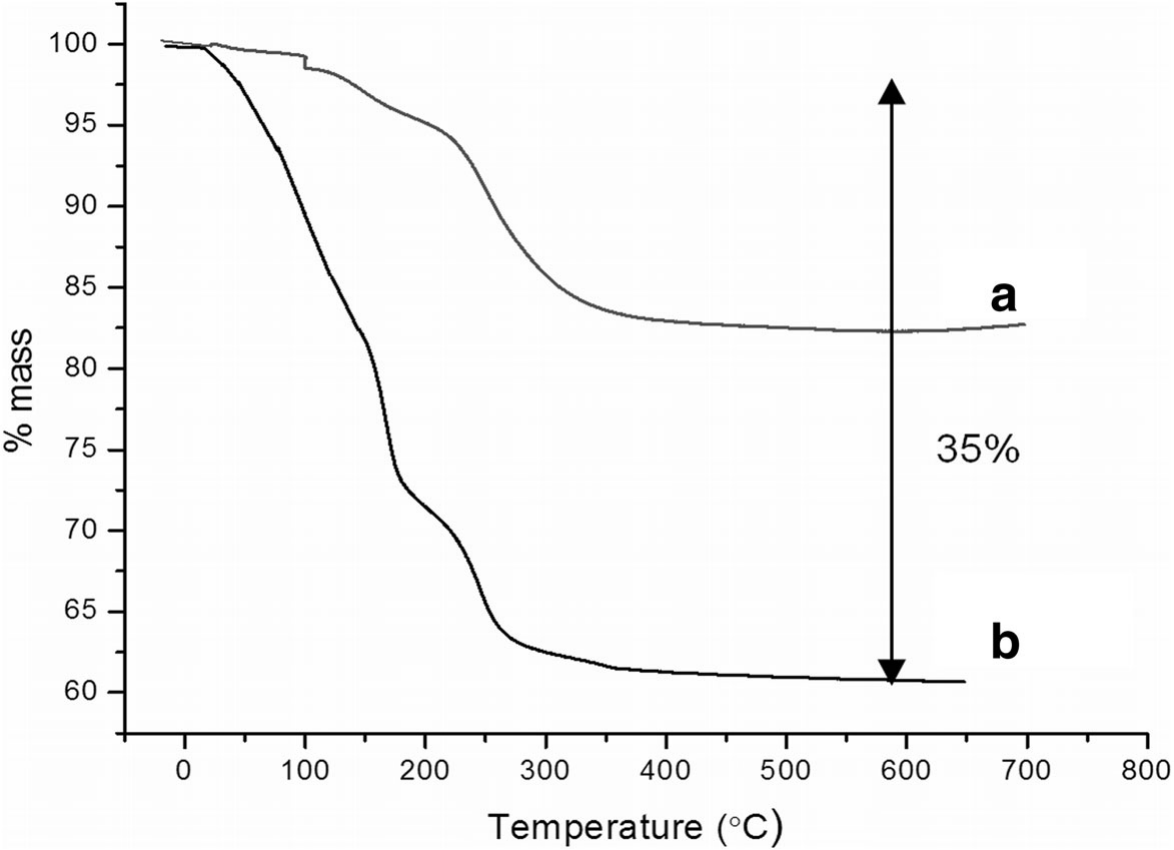
Thermograms of **a** SPIONs modified with sebacic acid and **b** SPIONs with A7R peptide

To estimate the amount of peptide conjugated to nanoparticles, thermogravimetric analysis was performed. Figure 5 presents a thermogram of peptide-modified (curve b) and unmodified (curve a) nanoparticles. The samples were heated under oxygen atmosphere to decompose the organic material from the surface of nanoparticles. On the basis of the thermogram, the quantity of peptides attached to the nanoparticles was calculated. We estimated that the peptide with linker constitutes ca. 35% of the conjugate mass, which gives the value 0.2 mg (0.24 μmol) A7R per 1 mg NPs.

### Cell cytotoxicity studies

**Fig. 6:**
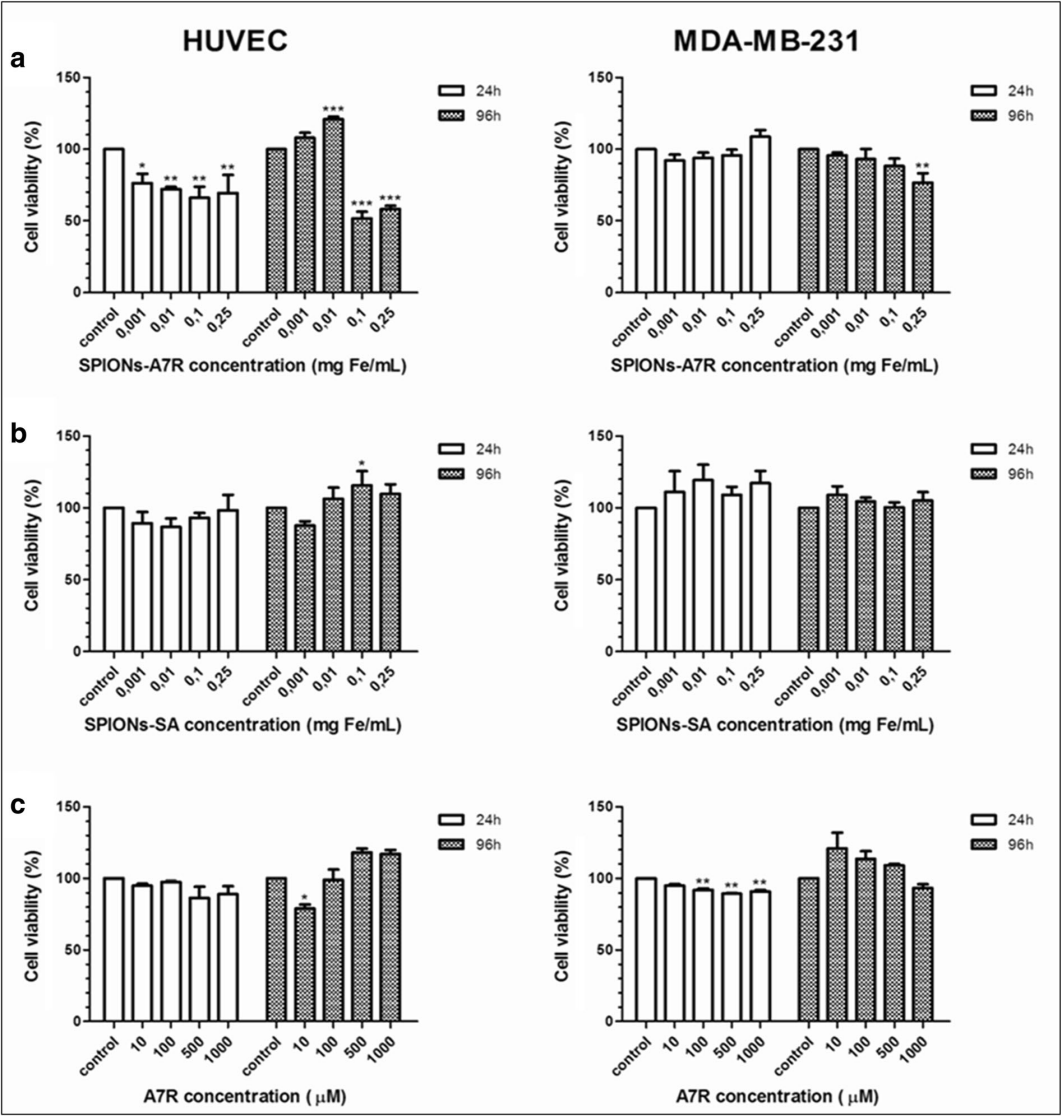
Influence of **a** SPIONs modified with A7R and **b** SPIONs modified with sebacic acid and **c** A7R peptide on HUVECs and MDA-MB-231 cell viability after 24 and 96 h of incubation. One-way ANOVA with Bonferroni’s multiple comparison test (concentration vs control).Values represent means ± SD determined from the results of three independent experiments, each performed in triplicate (*P < 0.05; **P < 0.01; ***P < 0.001)

Nanoparticles modified with sebacic acid and nanoparticles functionalized with A7R (conjugate) and A7R peptide were tested in vitro for the potential cytotoxic effect against two cell-line cancer (MDA-MB-231) and healthy (HUVEC) with NRP-1 expression. The study was carried out using MTS assay. The nanoparticles and conjugate were examined at iron concentrations between 0.001 and 0.25 mg/mL. The A7R peptide was examined at concentrations between 10 and 1000 μM. The viability of the cells was determined after 1- and 4-day incubation. The results are presented in Fig. 6.

Differences between two incubation times at various concentrations were analyzed by the two-way ANOVA with Bonferroni’s post-tests and presented in the Supporting Information (Fig. S5).

Cytotoxicity studies shows that SPIONs-SA do not display significant cytotoxic activity against cancer cells which remained more than 100–115% viable relative to control at all tested concentrations. A small cytotoxic effect is noticeable on cancer cell line in the case of higher concentration of iron in nanoparticles. Peptide A7R does not considerably affect the viability of both cell lines. However, after the conjugation of peptide with magnetic nanoparticles, cell viability decreased, especially on HUVECs cells—40–50% viability for higher SPIONs-A7R concentrations. It is worth mentioning that the process of removing medium with nanoparticles from HUVECs was very difficult, and in some cases, a part of cells could be removed or damaged, which may affect the final result.

Cell cytotoxicity studies indicated that SPIONs modified with A7R significantly reduced cell viability in comparison to cells that were exposed to the SPIONs with sebacic acid or A7R peptide at concentrations higher than 0.01 mg Fe/mL. One of possible explanations for this large decrease at high concentrations of SPIONs-A7R in cell viability may be that these nanoparticles are taken up by the cells more intensively as a result of A7R binding to the NRP-1 receptors on the surface of cells. It might be the alternative pathway of nanoparticle uptake which, beside the endocytosis, allows the entry of nanoparticles into the cell which could promote apoptosis. The specific interactions between similar peptide, KATWLPPR, conjugate to the gold NPs and NRP-1 on the surface of endothelial cells has been proved before (Bartczak et al. 2011). These results were supported by TEM images in which the trafficking of NRP-1 receptors with NPs were clearly observed. Nanoparticles are presumably mostly present on the external part of the cellular membrane and rarely internalized (Liu et al. 2013). Our results indicate that cytotoxicity is higher after 96 h of cell treatment with modified nanoparticles, endothelial cells showing lower viability than the cancer ones. Probably, SPIONs-A7R are degraded into iron ions in the lysosomes by hydrolyzing enzymes and it might lead to an imbalance in cell homeostasis (Singh et al. 2010). Free iron may provoke abnormal cellular responses including cytotoxicity, inflammatory processes, or oxidative stress (Fenton reaction). In general, cancer cells are more resistant for most of the factors than normal cells are. Recent evidences indicate that tumors have modified redox balance, deregulated redox signaling, and increased antioxidant ability (Panieri and Santoro 2016). It can be strongly implicated in resistance to treatment and also affects the viability of cancer cell line in this case. Interestingly, the cytotoxic effect of SPIONs-A7R in HUVEC is dose dependently counteracted by low concentrations of conjugated A7R after 96 h of incubation (Fig. 6a). This might indicate that A7R at low concentrations could act as a partial agonist of VEGF_165_ and mimics the prosurvival effect of VEGF_165_ on endothelial cells. This effect is also observed for A7R alone after 96 h of incubation (Fig. 6c). Therefore, depending on the concentration of endogenous VEGF_165_ secreted by HUVEC, A7R mimicking the exon 8 of VEGF_165_ can behave either as an antagonist or a weak agonist of VEGF_165_ (Hoyer and Bodekke 1993).

## Conclusions

In conclusion, we have demonstrated a successful synthesis of nanoparticles modified with the antiangiogenic and antitumor heptapeptide (A7R). A7R peptide was conjugated to SPIONs modified with sebacic acid via amide bond using EDC as a coupling reagent. Successful conjugation of A7R peptide to iron oxide-based nanoparticles was confirmed by complementary physicochemical analysis techniques (FTIR, SERS, SEM-EDS, TEM, and TGA). Cell cytotoxicity studies, against two cell lines (HUVEC and MDA-MB-231) indicated that SPIONs modified with A7R reduced HUVEC cell viability, at concentrations higher than 0.01 mg Fe/mL, in comparison to cells that were exposed to the SPIONs modified with sebacic acid or A7R peptide, what might be partially caused by a process of internalization. More in vitro studies are needed to prove internalization of SPIONs functionalized with A7R via receptor-mediated endocytosis. Development of NRP antagonists needs the discovery not only of specific and powerful drugs but also of biomarkers predicting the efficacy of the drug (companion diagnostics). As SPIONs could be used as platform not only for drugs but also for imaging agents, they are ideally positioned in this strategy of co-development. As imaging agents, they might also be used to assess the integrity of the blood brain barrier since NRP-1 is highly expressed in the brain. We plan to study these problems in our future work.

## Electronic supplementary material

ESM 1 (DOCX 865 kb).
