# Supplementary material for: Physicochemical properties and in vitro cytotoxicity of iron oxide-based nanoparticles modified with antiangiogenic and antitumor peptide A7R
Source: J Nanopart Res. 2017 Apr 26;19(5):160. doi: 10.1007/s11051-017-3859-x (PMC5406482; doi:10.1007/s11051-017-3859-x)
Supplement: Supplementary file 1 — (DOCX 865 kb). [file 11051_2017_3859_MOESM1_ESM.docx]

**Physicochemical properties and in vitro cytotoxicity of iron oxide-based nanoparticles modified with antiangiogenic and antitumor peptide A7R**

Anna Niescioruk^a^, Dorota Nieciecka^a^, Anna K. Puszko^a^ , Agata Królikowska^a^, Piotr Kosson^b^, Gerard Y. Perret^c^, Pawel Krysinski^a^, Aleksandra Misicka^a,b,*^

^a^ Faculty of Chemistry, University of Warsaw, Pasteura 1, 02-093 Warsaw, Poland

^b^ Department of Neuropeptides, Mossakowski Medical Research Centre, Polish Academy of Sciences, Pawinskiego 5, 02-106 Warsaw, Poland

^c^ Université Paris 13, Sorbonne Paris Cité, INSERM U1125, 74 rue Marcel Cachin, 93017 Bobigny, France

*Corresponding Author: e-mail: misicka@chem.uw.edu.pl

**Supporting Information**

**Synthesis and purification of A7R peptide**

The synthesis of the peptide ATWLPPR was carried out manually by the Fmoc solid-phase method, starting from Fmoc-Arg(Pbf)-Wang resin (0,5g; 0,39 mmol/g). Coupling reactions were conducted with 2,5 eq amino acid (Fmoc-Pro-OH, Fmoc-Pro-OH, Fmoc-Leu-OH, Fmoc-Trp(Boc)-OH, Fmoc-Thr(*t*Bu)-OH, Fmoc-Ala-OH), 2,5 eq TBTU, 2,5 eq 6-Cl-HOBt and 6 eq DIPEA in DMF (6 mL) for 2h. The completion of coupling reactions was monitored by Kaiser test (for primary amines) or chloranil test (for secondary amines). Fmoc group removals were performed with 20 % DIPEA in DMF (v:v) for 25 min (two steps: 5 + 20 min). After each deprotection step, the resin was washed: 3×DMF (6 mL), 3×IPA (6 mL) and 3×DMF (6 mL). After all coupling steps and final Fmoc deprotection, peptide-resin was washed: 3×DMF (6 mL), 3×DCM(6 mL), 3×Et_2_O(6 mL) and dried in vacuum. Cleavage of the peptide from the resin was performed by 5 mL a mixture of TFA: PhOH: H_2_O:TIS (88%: 5%:5%: 2%, v:v) for 2,5 h. TFA was removed under reduced pressure and peptide was precipitated with cold Et_2_O, isolated by centrifugation and lyophilized from water. Purification of peptide was carried out by preparative RP-HPLC (Shimadzu Instrument) with Phenomenex Jupiter 4µ Proteo 90A C_12_ column (250 x 21.2 mm) and monitored at 210 nm (UV-Vis detector SPD-20 A), using a linear gradient of water/acetonitrile solution containing 0,05% TFA. After purification we obtained 61,5 mg pure peptide. The scheme of A7R peptide synthesis is shown in Scheme S1.


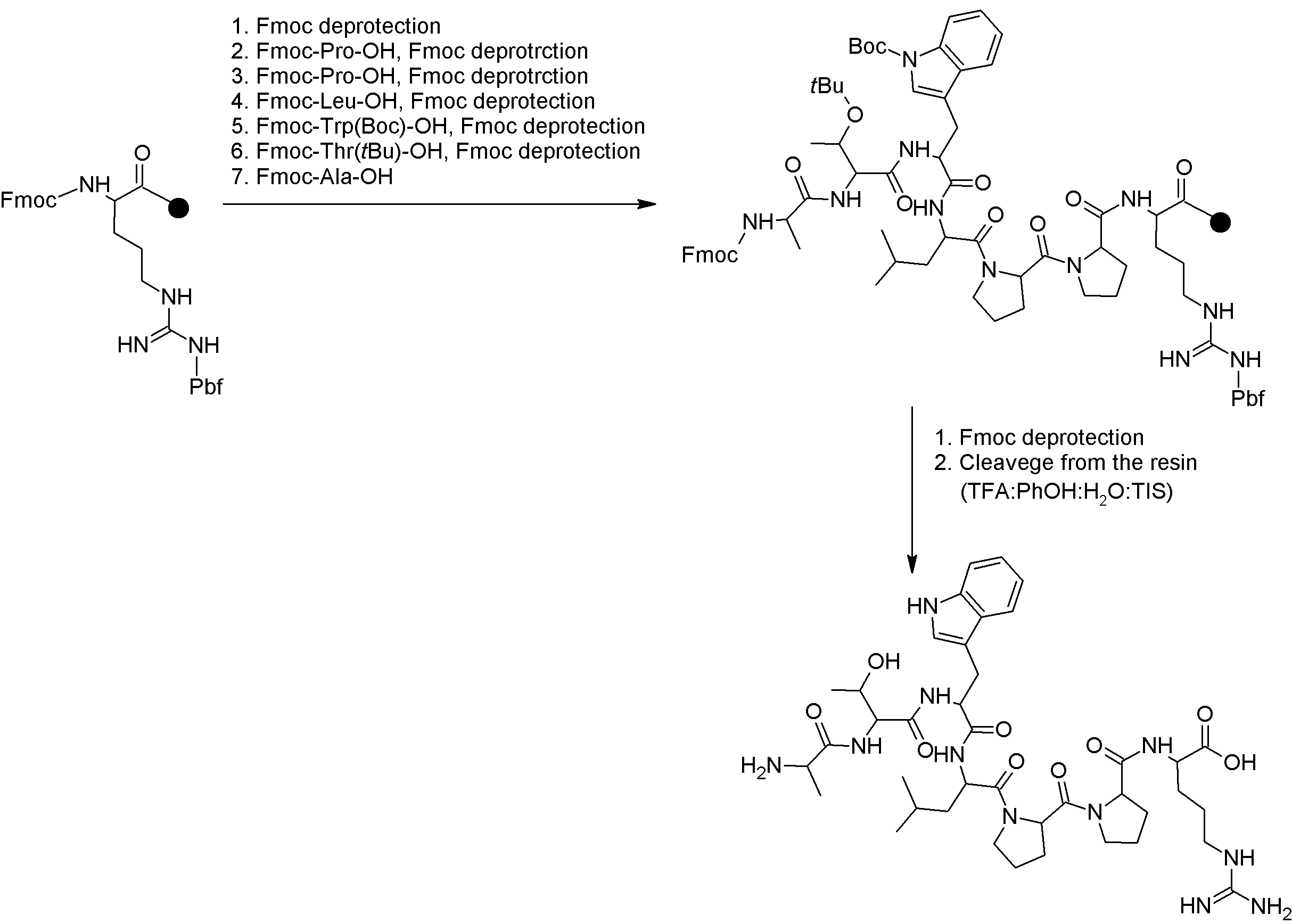


**Scheme S1.** Scheme of A7R synthesis.

**Analysis of A7R peptide purity using Reversed Phase-High Performance Liquid Chromatography (RP-HPLC)**

Purity of A7R peptide was checked by analytical RP-HPLC, which was carried out on the Shimadzu instrument with Phenomenex Jupiter 4µ Proteo 90A C_12_ column (250×4,6 mm) and a UV-Vis detector (SPD-20 A), using a linear gradient from 20% to 40% B in 20 min (A: 0,05 % TFA in water, B: 0,05 % TFA in acetonitrile, flow rate 1mL/min). UV detection was performed at 210 nm. The chromatogram for pure peptide is shown in Fig. S1 (t_r_ =7.612 min, purity 98 %).


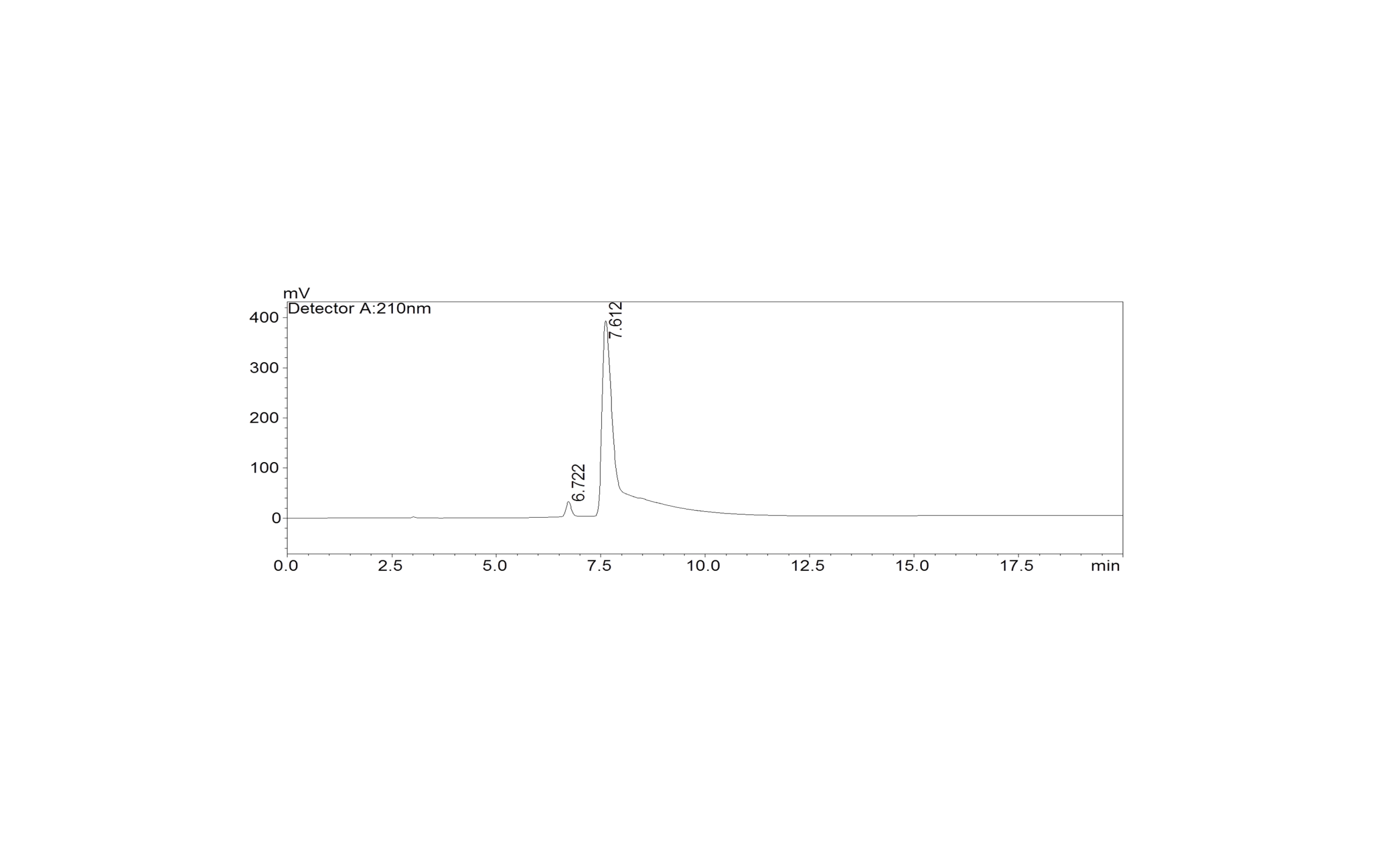


**Fig. S1.** The RP-HPLC chromatogram of purified A7R peptide

**Analysis of A7R structure using Reversed Phase-High Performance Liquid Chromatography (RP-HPLC) with Electrospray Ionization-Mass Spectrometry (ESI-MS)**

Peptide structure was confirmed by RP-HPLC-MS analysis which was carried out on the Shimadzu instrument LCMS 2010 EV with Phenomenex Jupiter 4µ Proteo 90A C_12_ column (250 ×2 mm), UV-Vis diode array detector (SPD-M20 A) and with electrospray ionization (ESI MS), using a linear gradient from 3% to 97% B in 40 min (A: 0,05 % TFA in water, B: 0,05 % TFA in acetonitrile, flow rate 0,3 mL/min) . UV detection was performed at 210 nm. Mass spectra was registered in positive ion mode. ESI-MS (m/z) calculated for C_40_H_61_N_11_O_9_:  839.47, Found: 840.42[M+H]^+^, 420.76[M+2H]^2+^


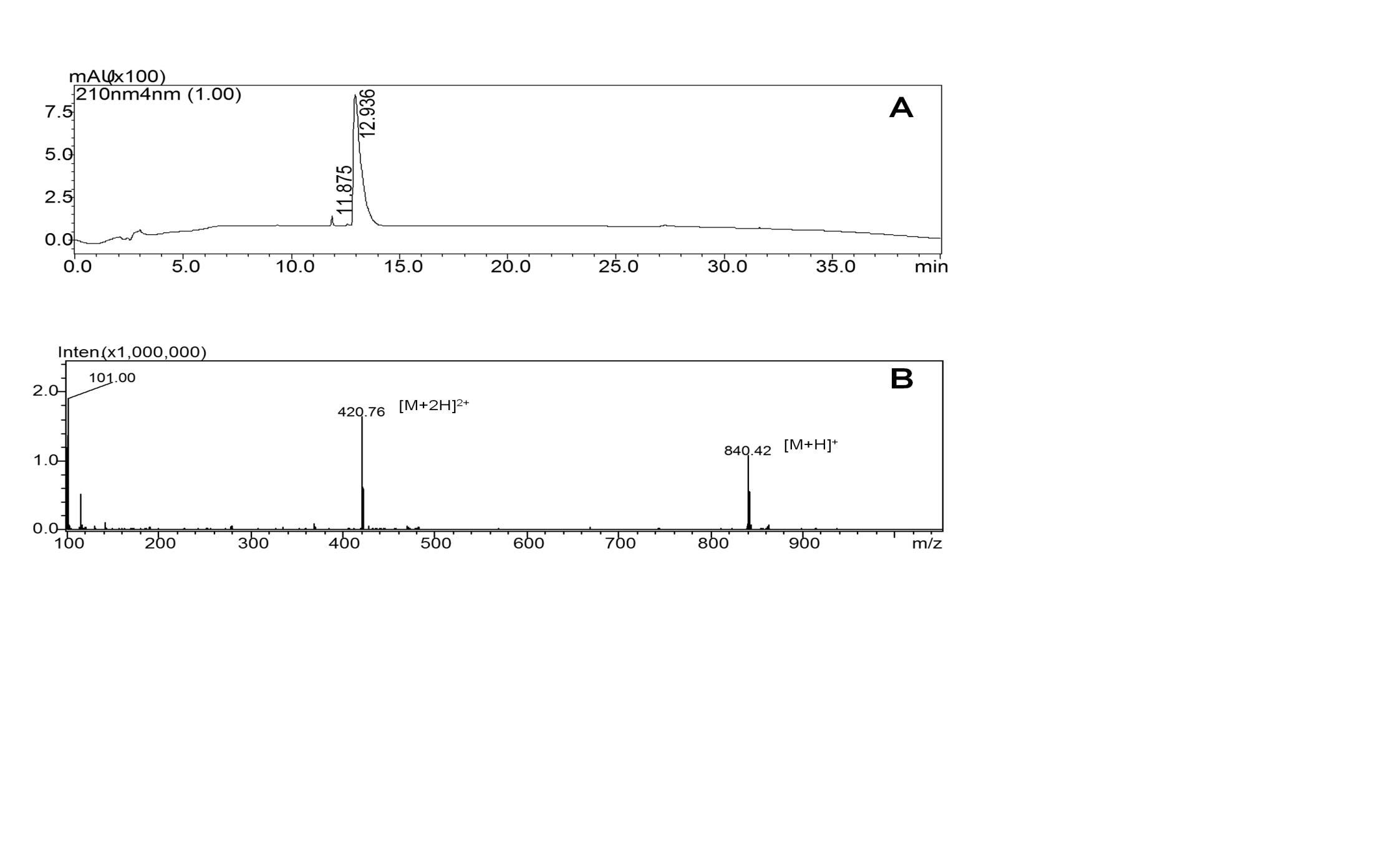


**Fig. S2.**The RP-HPLC MS analysis of pure A7R peptide (A) chromatogram for detection at 210 nm (B) MS spectrum of a component with t_R_=12,936 min

**Synthesis procedure of silver nanoparticles for surface-enhanced Raman scattering (SERS) spectra**

Silver colloid was prepared according to the procedure by Leopold and Lendl (2003), using hydroxylamine as a reducing agent. All used glassware was cleaned with *aqua regia* and then rinsed with plenty of deionized water. The UV-Vis spectrum of the silver colloid exhibited maximum at 410 nm. Based on the TEM imaging, the average silver nanoparticle diameter was determined to be around 20 nm.


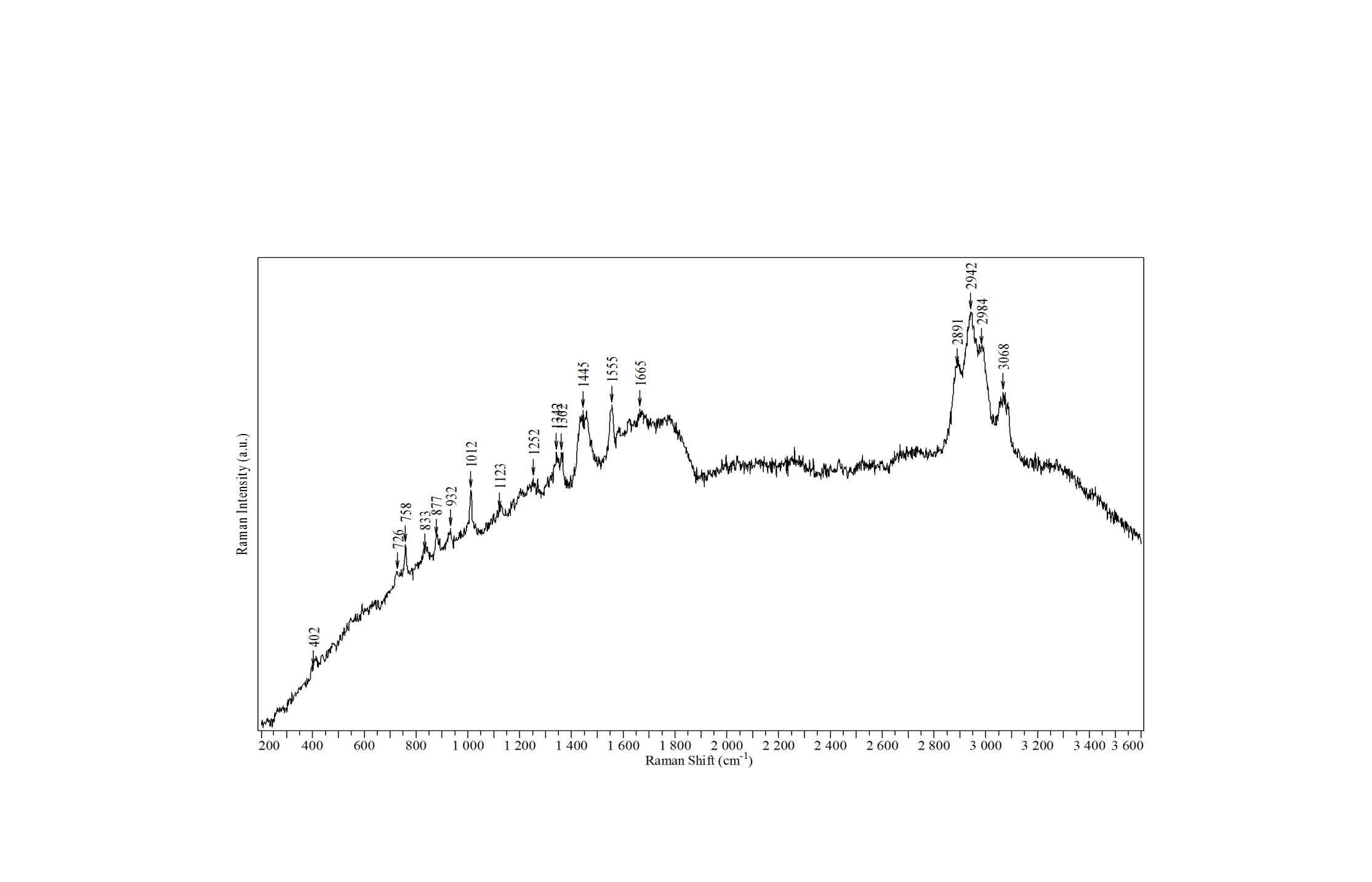


**Fig. S3** Normal Raman spectrum of solid A7R peptide, collected under 532nm laser excitation


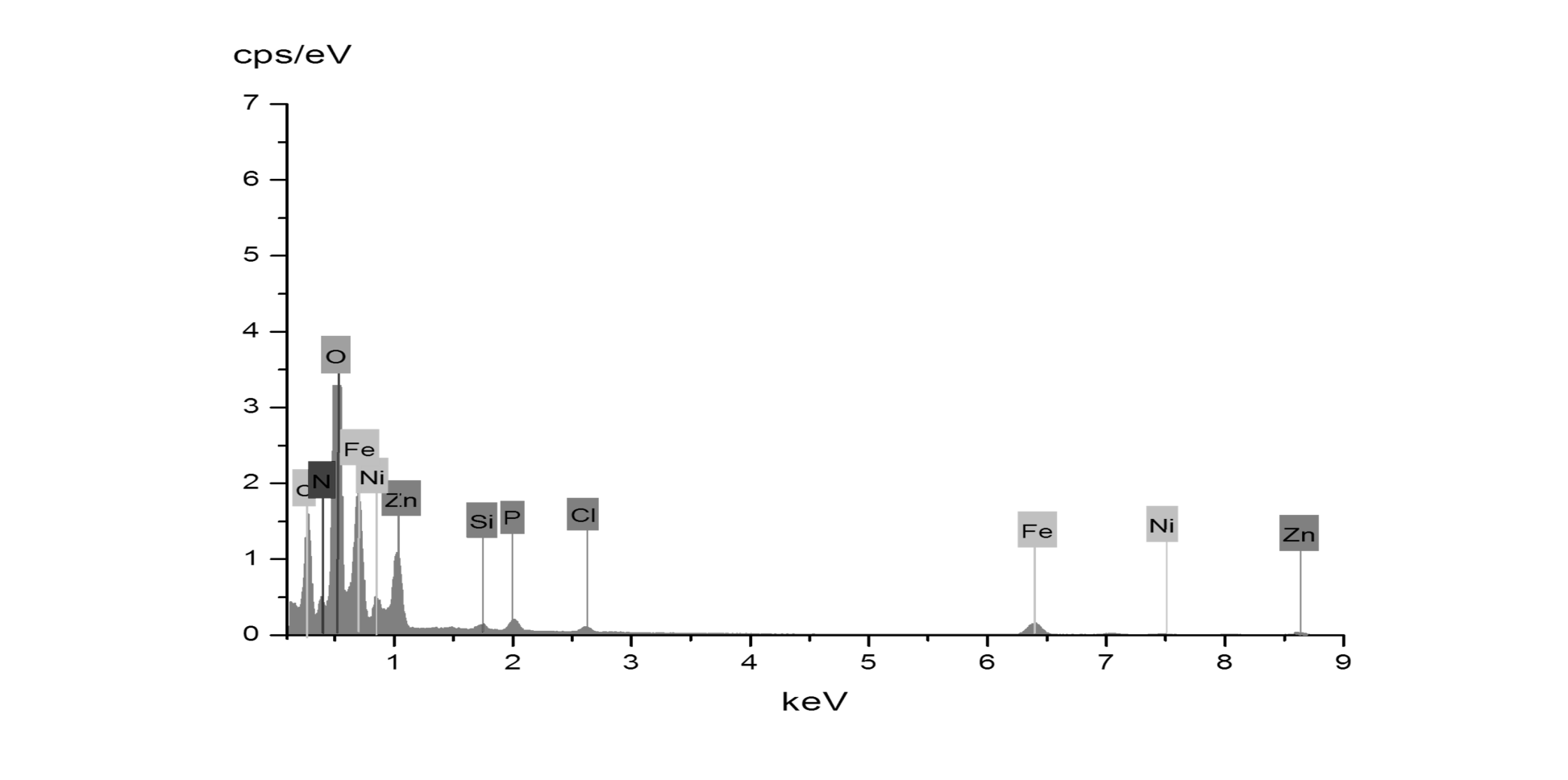
**Fig. S4** EDS spectrum of SPIONs modified with A7R peptide.


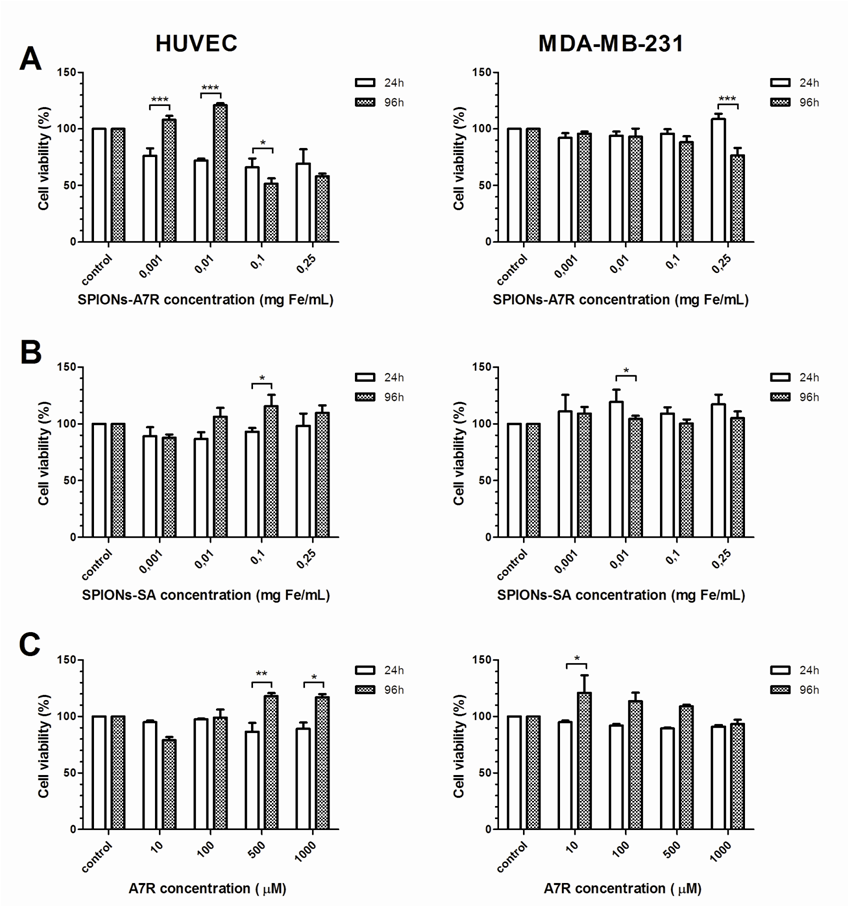


**Fig. S5** Influence of **(A)** SPIONs modified with A7R, **(B)** SPIONs modified with sebacic acid and **(C)** A7R peptide on HUVECs and MDA-MB-231 cells viability after 24 and 96 h of incubation. Differences between two incubation times at various concentrations were analyzed by the two-way ANOVA with Bonferroni’s post-tests. Values represent means ±SD determined from the results of three independent experiments, each performed in triplicate (*P<0.05; **P<0.01; ***P<0.001).

**REFERENCES**

Leopold N, Lendl B. (2003) A New Method for Fast Preparation of Highly Surface-Enhanced Raman Scattering (SERS) Active Silver Colloids at Room Temperature by Reduction of Silver Nitrate with Hydroxylamine Hydrochloride. J. Phys. Chem. B 107:5723-5727.doi: 10.1021/jp027460u
